# A Relationship-Centered and Culturally Informed Approach to Studying Misinformation on COVID-19

**DOI:** 10.1177/2056305120948224

**Published:** 2020-08-10

**Authors:** Pranav Malhotra

**Affiliations:** University of Washington, USA

**Keywords:** misinformation, COVID-19, mobile instant messaging services, WhatsApp

## Abstract

The panic and anxiety that accompanies a global pandemic is only exacerbated by the spread of misinformation. For COVID-19, in many parts of the world, such misinformation is circulating through globally popular mobile instant messaging services (MIMS) like WhatsApp and Telegram. Compared to more public social media platforms like Facebook and Twitter, these services offer private, intimate, and often encrypted spaces for users to chat with family members and friends, making it difficult for the platform to moderate misinformation on them. Thus, there is an enhanced onus on users of MIMS to curb misinformation by correcting their family and friends within these spaces. Research on understanding how such relational correction occurs in different parts of the globe will need to attend to how the nature of these interpersonal relationships and the cultural dynamics that influence them shape the correction process. Thus, as people increasingly use MIMS to connect with close relations to make sense of this global crisis, studying the issue of misinformation on these services requires us to adopt a relationship-centered and culturally informed approach.

## Introduction

On 19 March 2020, Indian Prime Minister Narendra Modi addressed the nation to discuss his government’s response to the COVID-19 pandemic and asked people to observe a voluntary 1-day lockdown 3 days later, foreshadowing longer lockdowns that would follow. During this speech, he also urged people to stand at their doorways or windows at 5 p.m. on the day of this lockdown and show their appreciation for essential workers by clapping and clanging utensils in unison. Soon, misinformation about how the noise generated by this practice would send out vibrations strong enough to weaken the virus was circulating all over the mobile instant messaging service WhatsApp (Mohan, 2020). Some groups in different regions of the country even took to the streets to make as much noise as possible, ignoring the need for social distancing (Sharma, 2020).

A global pandemic ratchets up panic and anxiety, which makes people even more susceptible to believing such misinformation, especially if it offers false hopes regarding treatments and the eradication of the virus. Here, misinformation refers to erroneous or false information that is not backed up by any evidence or expert opinion (Nyhan & Reifler, 2010). The unchecked spread of such health-related misinformation has been facilitated by a scattered media environment and can have grave consequences like worsening health conditions and increased deaths (Waisbord, 2020). Within this media environment, it is important to highlight the channels through which such falsehoods spread and how the nature of these channels can complicate efforts to curb misinformation. The example offered above is illustrative of the spread of misinformation through a channel that is not only popular in India but is utilized by a large section of the global population, namely mobile instant messaging services (MIMS) such as WhatsApp, WeChat, and Telegram. While not immensely popular in the United States, these services dominate technology use globally. The Facebook-owned service WhatsApp on its own has over 2 billion global users (Porter, 2020), while WeChat, which is a service that has a large user base in China, has over 1 billion users (Hollander, 2018). Considering these numbers, it is no surprise that stories like the one presented above, wherein false information on COVID-19 with no basis in science intersects with political propaganda, are spread through MIMS across the globe (Romm, 2020). Yet, there is not much scholarship on this issue of misinformation spread through MIMS. Furthermore, the technical features associated with MIMS as well as people’s use and perception of these services are different from social media platforms like Facebook and Twitter, which have received some scholarly attention regarding misinformation-related issues. Therefore, due to the global popularity of MIMS and the fact that such differences can influence how the spread of misinformation is curtailed on a particular channel (Vraga & Bode, 2017a), the issue of misinformation on these services, especially during a crisis like COVID-19, deserves to be looked at. These differences and how they inform the approach required to study this issue are discussed below.

## The Public/Private Distinction

Compared to social media platforms like Facebook and Twitter, which are viewed as more public and open, users view MIMS as private and intimate spaces, especially because they primarily use these services to communicate with close friends and family through both one-to-one and group chats (Boczkowski et al., 2018; O’Hara et al., 2014). Furthermore, technical features like end-to-end encryption, which are present on some of these services, reinforce the idea that they are private as the content being shared is ostensibly inaccessible to anyone other than the people participating in these chats.

This private nature of MIMS is problematic when it comes to the issue of misinformation. Typically, the content posted on social media is policed to a certain degree by the platforms themselves, through automated as well as human moderators (Roberts, 2019). Yet, the same is not true for MIMS. Technological interventions like algorithmic moderation are not possible on encrypted services like WhatsApp (Resende et al., 2019). Moreover, other technological solutions like limiting message forwarding have proven ineffective at curbing the spread of messages that are considered harmful (de Freitas Melo et al., 2019).

Thus, in the absence of technological solutions, misinformation correction on MIMS like WhatsApp is user-driven (Badrinathan et al., 2020). On these services, there is an enhanced onus on people to curb misinformation by correcting each other.

## Relational Correction

There is a wealth of scholarship on the effectiveness of different corrective messages in reducing misperceptions among people exposed to misinformation (see Lewandowsky et al., 2012). Recently, this topic has also been studied specifically within the context of digital spaces. Studies on the effectiveness of corrective messages on social media platforms reveal that misperceptions can be reduced through logic and humor-based corrections (Vraga et al., 2019), providing links to corroborating online sources (Vraga & Bode, 2017a), and corrections from reputable sources like official government agencies (Vraga & Bode, 2017b). Yet, much of this research is informed by the public nature of the social media platforms being studied. These studies consider how misperceptions may be reduced when an individual observes users they do not necessarily know correcting each other on their social media feed, which is a likely occurrence on platforms like Facebook and Twitter (Vraga & Bode, 2017b).

Observing such corrections can help in reducing the misperceptions engendered by the spread of misinformation on these platforms and research on this subject provides us with valuable insights. Yet, this does not easily apply to MIMS. Due to the private and intimate nature of these services, corrections on MIMS are instead more likely to occur between users who know each other. Scholarly work on social media and misinformation does find that people are more motivated to correct close friends and family members than strangers (Tandoc et al., 2019). Furthermore, a correction coming from a known other is more readily accepted than a correction from a stranger (Margolin et al., 2018). Yet, little is known about which corrective messages may work when it comes to what I call relational correction, which refers to instances where people correct family members and friends who share misinformation. In such situations, which are likely to occur on MIMS, in addition to the effectiveness of a corrective message in reducing misperceptions, various other factors that shape these interpersonal relationships may also come into play.

Thus, studying relational correction, which can help us understand how to curb misinformation regarding COVID-19 on MIMS, requires an approach that takes these factors into consideration.

## A Relationship-Centered and Culturally Informed Approach

Correcting a loved one or a dear friend is not simple. Such an act is informed by layers of relational history. Moreover, in relationships with status differentials and in cultural environments where correction is viewed as transgressive or threatening, deciding if one should engage in correction as well as considering which corrective message to utilize may be tumultuous and stressful choices to make. A model of relational correction therefore demands contextualization informed by theoretical perspectives from a research area like interpersonal communication, where relationship-related aspects are at the forefront. For example, utilizing social exchange theories, which focus on how individuals interact with people based on the costs and rewards associated with these interactions (Stafford, 2014), could help us examine when people are motivated to correct family members and friends who share misinformation. Meanwhile, politeness theory (Brown & Levinson, 1987), which outlines how people can have challenging conversations with others, can illuminate strategies for politely engaging in relational correction like emphasizing commonalities and in-group membership.

Given that misinformation is a global issue and MIMS are widely used in a variety of cultures, in addition to these relationship-related aspects, scholarship on this issue must also consider the cultural dynamics that influence misinformation propagation and correction. For instance, in my own research in India, I am interested in examining how young adults negotiate situations wherein their older relatives post misinformation about COVID-19 in family group chats on WhatsApp. In doing so, I have to consider how these young Indians negotiate the tension between traditional cultural ideals regarding deference toward elders, which may hinder them from correcting older relatives, and modern notions of reason and rationality (Van Wessel, 2012), which may encourage them to stop the flow of misinformation and call their elders out. Furthermore, the distribution of power within certain relationships may differ across cultures and is another aspect that scholars need to consider. For example, in cultures where familial relationships are characterized by rigid hierarchies based on gender and age, correcting an older male family member who shares misinformation may require a different strategy than correcting a peer. Recognizing the salience of these cultural values and norms is vital to identifying strategies that work within different cultures as people may want to curb misinformation on MIMS without contravening such values and norms.

It is important to clarify that advocating for this focus on how individuals may assume the responsibility for correcting each other should not be perceived as an attempt to let technology companies and governments off the hook for their failure in tackling misinformation. Scholarly work that critiques the response of these institutions to the spread of misinformation online remains immensely important, especially during a crisis like COVID-19. Furthermore, the arguments made here regarding the lack of technological solutions to curb misinformation on MIMS do not preclude the possibility of such solutions being developed in the future. Finally, although MIMS are the main focus here, scholars may also adopt this relationship-centered and culturally informed approach while studying other intimate and private seeming spaces on social media platforms. For instance, such an approach may be amenable to studying misinformation within small closed Facebook groups.

## An Approach *For* and *Of* This Moment

It may seem counter-intuitive to suggest that tackling the issue of misinformation on COVID-19 requires scholars to focus on micro-level phenomena like interpersonal relationships and cultural nuances when this pandemic affects the entire world and lays bare many broad structural issues. However, my choice to advocate for such an approach is informed by the nature of the social and cultural moment we are currently living through. As people practice social distancing around the world, they are utilizing mediated channels like MIMS to connect with family and friends. Thus, relational communication via such channels is only increasing in frequency. As more people engage in such communication during this pandemic, the likelihood of misinformation being shared through channels like MIMS increases. This makes it even more important to understand how misinformation operates within these private spaces. To do so, it is important for scholars to adopt a relationship-centered and culturally informed approach.

Drawing upon interpersonal communication theories, which have traditionally not been associated with an issue like misinformation, will enrich our understanding of how misinformation may be curbed through relational correction on MIMS. Consideration of how cultural aspects influence this practice is also a much-needed addition to the study of misinformation. Much of what we do know about misinformation comes from studies conducted within the United States that focus on social media platforms like Facebook and Twitter. Yet, misinformation, especially in relation to a global pandemic like COVID-19, circulates within and between many cultures through widely used MIMS. Thus, studying this issue in different regions requires that relational and cultural specificities be attended to. Even as the world faces the same challenge to stop the spread of COVID-19, a one-size-fits-all approach to studying misinformation related to the pandemic is not optimal.

## Conclusion

The public implications of examining how misinformation on COVID-19 may be curbed are abundantly clear when one considers the havoc such falsehoods can cause. By recognizing that in many parts of the world, such misinformation is being spread through MIMS, scholars and concerned parties can take the first step toward tackling this issue. Beyond this recognition, adopting a relationship-centered and culturally informed approach to study this issue will help everyone better understand how the spread of misinformation through MIMS can be curtailed. Such knowledge is not only relevant to understanding the misinformation environment during this COVID-19 crisis. Rather, as these services continue to grow in global popularity, it will also help us understand how to tackle misinformation during future crises that may follow.
